# Evaluating the Effectiveness of sEMG Biofeedback for Posture Training and Scoliosis Management

**DOI:** 10.1155/bmri/6391772

**Published:** 2026-04-27

**Authors:** Yiu-Hong Wong, Mei-chun Cheung, Qi-Wen Emma Lei, Joanne Yip

**Affiliations:** ^1^ School of Fashion and Textiles, The Hong Kong Polytechnic University, Hung Hom, Kowloon, China, polyu.edu.hk; ^2^ Department of Social Work, The Chinese University of Hong Kong, Hong Kong, Shatin, New Territories, China, cuhk.edu.hk

**Keywords:** AIS, biofeedback training, muscle imbalance, scoliosis, sEMG

## Abstract

Digital devices, particularly smartphones and tablets, are used by the vast majority of the global population. This usage often causes the head to tilt forward, placing significant strain on the neck and shoulders. While this posture adversely affects healthy individuals, it is particularly problematic for scoliosis patients, exacerbating spinal deformation. Therefore, this study evaluates the effectiveness of a 30‐session surface electromyography (sEMG) biofeedback posture training program for managing spinal curvature progression in adolescents with mild scoliosis. The program is aimed at reducing imbalanced paraspinal muscle activity and controlling curvature progression. Prior to training, a significant muscle activity imbalance was observed. Posttraining, however, muscle activity becomes more balanced, with significant improvements noted in the latissimus dorsi and thoracic erector spinae muscles. It also demonstrates that the training may assist in stabilizing scoliosis progression. These findings suggest that sEMG biofeedback posture training can be an effective intervention for adolescents with mild scoliosis. However, further research is needed to confirm the findings and explore the intervention′s long‐term effects.

## 1. Introduction

The pervasive use of smartphones and tablets in daily life has inadvertently fostered a culture of poor posture, especially among adolescents [1, 2]. Habitual forward head tilt during device use places undue stress on the cervical spine, exacerbating musculoskeletal strain and spinal misalignment [3]. This biomechanical compromise destabilizes the spine by disrupting muscle forces and intra‐abdominal pressure dynamics [4, 5].

Adolescent idiopathic scoliosis (AIS), a prevalent condition among youths during pubertal growth spurts [6], is defined by a lateral spinal curvature exceeding 10° measured by Cobb′s method. The etiology of AIS is multifaceted, involving a complex interplay of genetic, environmental, hormonal, and neuromuscular factors [7–9], including abnormal growth patterns, asymmetrical muscle development, and neurologic abnormalities. While its precise cause remains elusive, early intervention using orthoses like hard braces—particularly when the Cobb angle is below 20°—can halt curvature progression [10, 11]. Despite their efficacy in providing corrective pressure, traditional rigid braces present limitations such as discomfort and social inconvenience [12, 13]. Furthermore, orthotic treatment success depends significantly on the patient′s postural awareness and commitment to self‐correcting spinal misalignment [14–16].

Historically, management for mild AIS (Cobb angle < 20°) prioritized passive observation [17]. However, 24%–58% of observed patients progress to bracing during rapid growth phases [18, 19]. Consequently, modern international consensus, including the 2016 SOSORT guidelines, advocates for an active, conservative approach for mild curves (10°–25°) in growing adolescents. Rather than a pure “wait and see” approach, guidelines recommend physiotherapeutic scoliosis‐specific exercises (PSSEs) to improve muscle symmetry and lower the risk of progression [10, 20]. Recent meta‐analyses and controlled trials have confirmed that structured exercise programs, such as the Scientific Exercise Approach to Scoliosis (SEAS) and Schroth methods, are effective in stabilizing Cobb angles and preventing severe progression in mild AIS without the immediate need for bracing [21–23].

Several alternative treatments address issues associated with traditional management. Durmała et al. [24] proposed active 3D correction, mobilizing the primary curve toward correction by emphasizing thoracic kyphotization and/or lumbar lordotization. Lehnert‐Schroth [25] developed an exercise regimen centered on specific rotational angular breathing. Romano et al. [26] proposed a method based on scoliosis‐specific active self‐correction (SEAS), performed without external aids and integrated into functional exercises. These approaches demonstrate that proficient motor control enables immediate, temporary spinal adjustments. However, they typically require patients to execute specific movements or maintain fixed postures, with feedback usually provided by an instructor. Given their focus on enhancing motor control, incorporating a biofeedback system could potentially augment their effectiveness.

While outcomes vary, these alternative treatments generally aim to improve spinal alignment and reduce curvature progression. Drawing on these insights, biofeedback is proposed as a complementary tool rather than an exercise itself [27]. It provides real‐time data on physiological functions, increasing patient awareness of postural habits and muscle activation patterns. Integrating biofeedback with therapeutic exercises may enable more precise movement control, leading to more effective and sustained spinal posture corrections [28].

Building on these strategies, Cheung et al. [29] developed a novel posture training program integrating surface electromyography (sEMG) biofeedback to independently monitor and train spinal muscle groups. Preliminary findings suggest that this biofeedback‐facilitated training can mitigate imbalanced paraspinal muscle activity [29, 30]. This study therefore is aimed at evaluating the impact of a 30‐session sEMG biofeedback posture training program on paraspinal muscle balance and spinal curvature management in adolescents with mild scoliosis. We hypothesize that participants will exhibit more balanced muscle activity and stabilized curvature progression postintervention. Additionally, the study explores the influence of specific paraspinal muscles on scoliosis trajectory. A preprint of this manuscript has previously been published (10.21203/rs.3.rs-4954337/v1).

## 2. Methods

### 2.1. Participants

A total of 25 adolescents (female = 16, male = 9), between 9 and 14 years old (mean = 11.88, SD = 0.971), were selected as subjects through a school screening program. During this program, the angle of trunk rotation (ATR) in the thoracic and lumbar regions was measured by using a scoliometer during Adam′s forward bend test. Subjects with an ATR greater than 4° underwent further evaluation, which included EOS imaging of the entire spine. To minimize measurement bias, the Cobb angles before and after the intervention were measured by an independent medical physician at an external private clinic. This assessor was blinded to the participants′ biofeedback posture training results. The subjects with a Cobb angle that ranges from 10° to 20° were invited to participate in this study. Details of their profiles are provided in Table 1. Participation was voluntary, with informed consent obtained from the adolescents themselves and written informed consent from their parents. Ethics approval was received from the ethics committee of the university of the authors.

**Table 1:** Participant profile and their spinal deformity angles before and after biofeedback posture training.

Subject no.	Age	Sex	Height (cm)	Weight (kg)	BMI	Major curve angle (before training)	Major curve angle (after training)	Difference
1	12	M	165	57.1	21	11.7°	20.7°	9°
2	12	M	153	41	15.9	17.6°	21.7°	4.1°
3	12	F	153	37.3	17.5	21.1°	26.9°	5.8°
4	13	F	155	43.5	18.1	16.3°	6°	−10.3°
5	11	F	150	39.1	17.4	16.2°	20°	3.8°
6	13	F	167	49	17.6	17°	16°	−1°
7	11	F	143	31.4	15.3	13.2°	7.6°	−5.6°
8	11	F	159	38.9	15.4	14.7°	19°	4.3°
9	9	F	146	37.4	17.7	15°	26°	11°
10	12	M	152	43	18.6	17.5°	17°	−0.5°
11	11	F	153	38.5	16.4	14.4°	11°	−3.4°
12	12	M	167	46.9	17.1	16°	9.7°	−6.3°
13	11	M	155	59.2	24.6	12.5°	6°	−6.5°
14	12	F	158	37.5	15.3	17.2°	18°	0.8°
15	13	M	168	51.8	18.4	13.9°	12°	−1.9°
16	11	M	158	59.8	23.8	13.5°	14°	0.5°
17	13	F	162	39.4	15	11.3°	17°	5.7°
18	12	F	141	34.2	17.2	19°	25°	6°
19	12	F	149	36.4	16.4	12.6°	9°	−3.6°
20	12	F	142	40.6	20.1	12.4°	9°	−3.4°
21	12	F	160	46.9	18.3	12°	15.3°	3.3°
22	14	F	160	43.3	16.9	15°	9.9°	−5.1°
23	12	M	164	56.2	20.9	11°	10°	−1°
24	12	M	166	53.5	19.4	13.2	11.3	−1.9
25	12	F	155	40.0	16.6	12.3	13.4	1.1

### 2.2. sEMG Measurement Prior to and Posttraining

**Figure 1:**
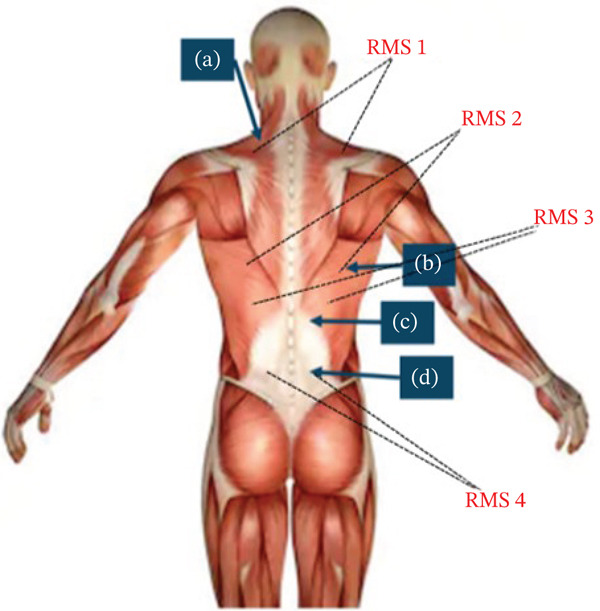
sEMG monitoring of pairs of spinal muscles: (a) trapezius, (b) latissimus dorsi, (c) thoracic erector spinae, and (d) lumbar erector spinae muscles [31].

The activity of the spinal muscles was measured by using a preamplifier sensor, MyoScan (Model T9503M; Thought Technology Ltd., Canada), and a data acquisition system, FlexComp Infiniti (Model T7555M; Thought Technology Ltd., Canada). The sEMG electrodes were positioned on the paraspinal muscles, specifically the trapezius, latissimus dorsi, thoracic erector spinae, and lumbar erector spinae muscles in pairs to assess muscle activity along the spine during habitual sitting postures (see Figure 1). The skin of the subject was cleaned with an alcohol wipe to reduce impedance to less than 5 k*Ω*, which was verified by an impedance test built into the BioGraph Infiniti software (Thought Technology Ltd., Canada). The sEMG signals were sampled at a rate of 2048 Hz with a 10–500 Hz band‐pass filter and a 60 Hz notch filter to eliminate artifacts and noise.

### 2.3. Biofeedback Posture Training Setup

All of the participants underwent an assessment before and after the biofeedback posture training. During each assessment, they were asked to sit for 3 min in a relaxed state to measure their muscle activity via sEMG signals. The baseline measurement protocol was administered by using BioGraph Infiniti, with measurements taken three times for 3 min each time.

Each adolescent completed 30 training sessions, which were delivered once a week over a period of approximately 6 months. Prior to each training session, an impedance check was performed after the placement of the sEMG electrodes. During posture training, the participants were instructed to sit in their ideal position and relax the four pairs of paraspinal muscles for 5 min. Videos on the biofeedback screen were shown to them when the sEMG values of the paraspinal muscles and the root mean square (RMS) sEMG ratio of the pairs of paraspinal muscles fell below the threshold of specific individual requirements. The aim of the training was to reduce the RMS sEMG ratio between the left and right sides of the four pairs of muscles toward 1 and the individual sEMG values of each of those muscles to less than 5 *μ*V. The exact threshold was individually tailored. Specifically, the sEMG threshold for video playback was set to 80% of the average sEMG value achieved by the individual in their previous training session. Increased relaxation of the paraspinal muscles and balance of the muscle activity of the four pairs of paraspinal muscles resulted in an increase in the video playback time. This posture training routine was repeated five times in each session, with a 2‐min rest given between each repetition. Each training session lasted approximately 60 min. The sEMG biofeedback posture training protocols were developed by using BioGraph Infiniti.

### 2.4. Statistical Calculations

All of the statistical analyses were performed using SPSS Statistics 25 for Windows (IBM Corp., Armonk, New York) and RStudio 2023.12.1+402 “Ocean Storm” Released for Windows. The ratio was calculated for the RMS of the sEMG signals from the four pairs of paraspinal muscles using the following formula:

r=RMSconvexRMSconcave.

Since each pair of muscles was measured three times, the mean of the ratios for each pair was subjected to one‐sample *t*‐tests to identify any imbalance (deviation from the test value of 1) in sEMG signals over the trapezius, latissimus dorsi, thoracic erector spinae, and lumbar erector spinae muscles, before and after the biofeedback training. A one‐sample *t*‐test was also performed for the mean of the ratios. A repeated measures analysis of variance (ANOVA) was conducted to compare the RMS sEMG ratios of the four pairs of muscles before and after the training, followed by a post hoc pairwise comparison if the multivariate results were significant. The level of significance was set at 0.05. Then, a survival analysis was conducted by using the Cox proportional‐hazards model to evaluate the effect of paraspinal muscle asymmetry on the progression of AIS. In this model, indexes are used instead of RMS sEMG ratios in order to provide a better quantitative description of the muscle asymmetry. The indexes are calculated as follows:

Index=1−1−r1+r

where *r* is the RMS sEMG ratio of each pair of paraspinal muscles.

### 2.5. Statistical Results

Prior to training, the one‐sample *t*‐test results indicated a significant deviation from the ratio of 1 in the RMS sEMG ratios over the pairs of thoracic erector spinae muscles (mean = 1.990, *t*(24) = 2.381, *p* < 0.05) and lumbar erector spinae muscles (mean = 1.184, *t*(24) = 2.093, *p* < 0.05) in the sitting posture. The results of the nonparametric Wilcoxon signed‐rank test also showed a similar significant difference for the thoracic erector spinae and lumbar erector spinae muscles (*p* < 0.05). In contrast, after training, only the RMS sEMG ratio over the pair of thoracic erector spinae muscles (mean = 1.370, *t*(24) = 2.301, *p* < 0.05) significantly deviates from the ratio of 1 in the sitting posture. The results of the nonparametric Wilcoxon signed‐rank test also showed a similar result in which the thoracic erector spinae muscles are significantly different from 1 (*p* > 0.05), whereas the other three muscle pairs are not significantly different from 1.

A repeated measures ANOVA was used to examine the main effect of the intervention (prior to and posttraining) on the relative balance in sEMG activity across the four pairs of paraspinal muscles by comparing the RMS sEMG ratios before and after the training. Additionally, the interaction between intervention and pairs of muscles was also examined.

Regarding the main effect of the intervention, a multivariate test showed that there is no significant difference before and after the intervention, that is, prior to and posttraining (*F*(1, 24) = 1.971, *p* > 0.05). In contrast, the multivariate test showed that the main effect of the pairs of muscles is significantly different (*F*(3, 72) = 3.201, *p* < 0.05, partial *η*2 = 0.118).

For the intervention × muscle pair interaction, the multivariate test showed that the interaction is significant (*F*(3, 72) = 3.304, *p* < 0.05, partial *η*2 = 0.121). A post hoc pairwise comparison revealed that the posttraining RMS sEMG ratio of the latissimus dorsi muscles is significantly smaller (M = 1.184, *t*(24) = 2.079, *p* < 0.05) than that at baseline (M = 1.990). The results of Student′s paired *t*‐test also showed a significant difference for the latissimus dorsi (*t*(24) = 1.803, *p* < 0.05) and thoracic erector spinae (*t*(24) = 1.929, *p* < 0.05) muscles.

A survival analysis was conducted using the Cox proportional‐hazards model with the posttraining sEMG index of all four pairs of muscles. The model showed that the beta coefficient of the posttraining sEMG index of the thoracic erector spinae muscles in the sitting posture is negative (*β* = −6.685, *p* < 0.05). Due to the small sample size, the likelihood ratio test was implemented. The Cox proportional‐hazards model is significant (*λ* = 11.09, *p* = 0.03) on 4 degrees of freedom.

## 3. Discussion

**Figure 2:**
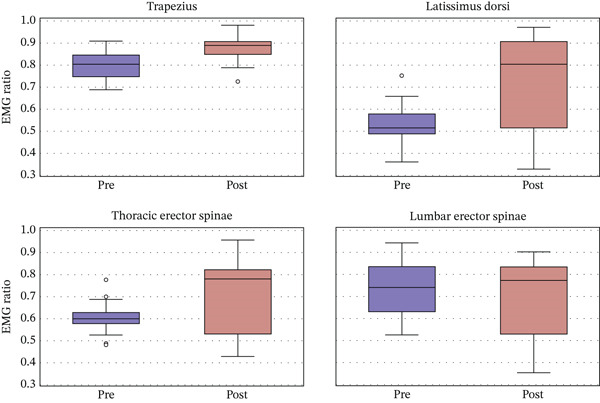
RMS sEMG ratios before and after the biofeedback posture training.

The RMS of the sEMG ratios presented in Figure 2 provides evidence of imbalance in some of the paraspinal muscle activity among the 25 adolescents diagnosed with AIS prior to our posture training. This imbalance is particularly noticeable in the latissimus dorsi and thoracic erector spinae muscles. To elaborate, the average RMS sEMG ratios for these specific pairs of muscles were found to be significantly larger than 1. This statistical finding suggests a clear imbalance in the activity of these two paraspinal muscles before the adolescents underwent our posture training. Ratios larger than 1 indicate a relatively higher level of muscle activity on the convex side of the spine compared to the concave side. Furthermore, the observed asymmetry of the latissimus dorsi muscles is consistent with the results of previous research conducted on AIS patients [29]. These studies reveal a larger average RMS sEMG value on the convex side of the trapezius muscles, which is attributed to a lower level of bioelectric activity on the concave side of the muscles [32]. This agreement with previous findings further validates the presence of muscle asymmetry in AIS patients and underscores the need for targeted interventions to address the imbalance.

Following a regimen of 30 sessions of biofeedback posture training, the RMS sEMG ratios of the paraspinal muscles of the tested subjects approached 1. This suggests a shift toward more symmetric activity of the pairs of muscles compared to the measurements taken prior to the training. Notably, significant improvements were observed in the muscle activity of the latissimus dorsi and thoracic erector spinae muscles posttraining. Given that the biofeedback posture training relies on sEMG signals as a feedback mechanism for monitoring posture, it is plausible that the training would have had a significant impact on the sEMG signals posttraining. Imbalanced paraspinal muscle activity is widely recognized as a key risk factor for the progression of spinal curvature [33]. Therefore, to determine the benefits of the biofeedback posture training, another more objective outcome measure, namely, spinal deformity, was assessed both before and after the training.

We hypothesized that a reduction in the imbalance of paraspinal muscle activity could potentially have beneficial effects in controlling the progression of the spinal curvature over time. It is important to note that due to potential human error, there could be a 5° margin of error when measuring the Cobb angle. Therefore, subjects with less than 5° of progression were considered to show no progression in spinal deformity. While significant regression of the curvature was not observed on average, preventing progression remains the primary clinical objective for mild AIS. According to recent clinical guidelines and meta‐analyses, active conservative management, such as specific exercises and posture training, is deemed highly successful if it maintains curve stability and improves muscle symmetry during pubertal growth spurts, thereby delaying or avoiding the need for bracing [10, 20, 21].

In line with our hypothesis, the RMS sEMG ratio of the latissimus dorsi muscles in the sitting posture of subjects with progression of scoliosis (M = 0.892) is smaller (further away from 1) than that of those without progression (M = 1.050) when comparing the RMS sEMG ratios of the paraspinal muscles after training. Additionally, the lumbar erector spinae muscles in a sitting posture of the subjects with progression of scoliosis (M = 1.619) are less balanced (further than 1) than those without progression (M = 1.103). These results show that by correcting the asymmetry of the paraspinal muscles, spinal deformity could potentially be mitigated. The sample *t*‐test showed that the changes in the curvature angles of the spine are significantly less than 5° (*t*(24) = −4.600, *p* < 0.05), thus indicating that the progression of scoliosis is effectively controlled during the posture training.

A limitation of this study is the relatively small sample size (*n* = 25). While significant main effects were observed, the limited sample size restricts the statistical power to detect small‐to‐moderate interaction effects between different muscle groups and the training intervention. Furthermore, without a nonintervention control group, we cannot definitively conclude that the stabilization of the Cobb angle was solely due to the biofeedback training rather than the natural growth trajectory of mild AIS. Future studies utilizing larger cohorts and randomized controlled designs are needed to validate these findings and allow for adequately powered subgroup analyses.

## 4. Conclusion

The findings in this study suggest that the use of sEMG biofeedback in posture training can effectively reduce the imbalance of paraspinal muscle activity and manage the progression of spinal curvature in adolescents with mild scoliosis. The results show significant improvements in muscle activity, particularly in the latissimus dorsi and thoracic erector spinae, following the 30‐session training program. The study also finds that the RMS sEMG ratios of all of the paraspinal muscles of the tested subjects approach 1 posttraining, thus indicating a shift toward more balanced muscle activity. This suggests that the biofeedback posture training can potentially have beneficial effects in controlling the progression of the curvature over time. However, further research with a larger sample size is needed to confirm these findings and explore the long‐term effects of this intervention on the quality of life of adolescents with scoliosis.

## Author Contributions

Y‐H.W., M‐C.C., and J.Y. contributed to drafting the manuscript and reviewing the content. M‐C.C. and J.Y. contributed to the conception and design of the research. Y‐H.W. and M‐C.C. conducted the analyses. Y‐H.W. and M‐C.C. contributed to formal analysis and validation. M‐C.C. and J.Y. contributed to project administration and supervision. Y‐H.W., M‐C.C., and Q‐W.E.L. contributed to conducting and data collection of the research. All authors reviewed the manuscript.

## Funding

This research was supported by multiple sources, including a research fund from the Lee Hysan Foundation to the Hong Kong Polytechnic University under the project “Non‐Surgical Treatments for Adolescents With Spinal Deformity” (R‐ZH3Y, J.Y.), a Research Postgraduate Scholarship from the Hong Kong Polytechnic University (Y‐H.W.), a grant from the RGC Collaborative Research Fund for the project “Optimising Spinal Curvature Corrective Outcomes in Adolescent Idiopathic Scoliosis: An Investigation into Spinal Flexibility, Biomechanical Behaviour and Predictive Modelling” (Grant Number C5058‐24G, J.Y.), and a grant from the Research Grants Council of the Hong Kong Special Administrative Region, China, to the Chinese University of Hong Kong (Grant Number CUHK 14607519, M.C.).

## Disclosure

A preprint of this manuscript has previously been published (10.21203/rs.3.rs-4954337/v1).

## Conflicts of Interest

None of the authors have a conflict of interest to disclose

## Data Availability

The data that support the findings of this study are available from the corresponding author upon reasonable request.
